# Role of Functional Foods and Nutraceutical Compounds in Alleviating Polycystic Ovary Syndrome: A Narrative Review

**DOI:** 10.7759/cureus.106497

**Published:** 2026-04-05

**Authors:** Pooja Singnale, Debasis Sasmal, Surya Goud S. Chukkala, Monica Chilumula, Raghavendra Pandurangi, Soibam Peter Singh, Mahesh Kumar Mummadi, Ramesh Gondru

**Affiliations:** 1 Home Science, Krishi Vigyan Kendra, Anjaw, Indian Council of Agricultural Research (ICAR)-Research Complex for North Eastern Hill (NEH) Region, Basar, IND; 2 Aquaculture, Krishi Vigyan Kendra, Anjaw, Indian Council of Agricultural Research (ICAR)-Research Complex for North Eastern Hill (NEH) Region, Basar, IND; 3 Public Health Nutrition, Indian Council of Medical Research (ICMR) National Institute of Nutrition, Hyderabad, IND; 4 Maternal and Child Health Nutrition, Indian Council of Medical Research (ICMR) National Institute of Nutrition, Hyderabad, IND; 5 Medical Research, Academy of Scientific and Innovative Research, Ghaziabad, IND; 6 Social Science, Krishi Vigyan Kendra, Anjaw, Indian Council of Agricultural Research (ICAR)-Research Complex for North Eastern Hill (NEH) Region, Basar, IND; 7 Clinical Epidemiology, Indian Council of Medical Research (ICMR) National Institute of Nutrition, Hyderabad, IND; 8 Food Chemistry, Indian Council of Medical Research (ICMR) National Institute of Nutrition, Hyderabad, IND

**Keywords:** curcumin, flaxseed, inositol, n acetylcysteine, omega 3 fatty acid, polycystic ovary syndrome (pcos), pumpkin seeds, seed cycling, sesame seeds, sunflower seed

## Abstract

Polycystic ovary syndrome (PCOS) represents one of the most common endocrine disorders among women of reproductive age. It is often associated with hormonal imbalance, metabolic disturbances, and reproductive complications, which together contribute to an elevated risk of long-term cardiometabolic disorders. Limitations and side effects of conventional pharmacotherapies have prompted growing interest in nutrition-based adjunct strategies targeting core PCOS pathophysiology. To narratively synthesize mechanistic rationale and human clinical evidence on dietary seeds and selected nutraceuticals relevant to PCOS, with emphasis on metabolic and reproductive endpoints of interest to nutrition and obstetrics/gynecology practice. A targeted literature search was conducted in PubMed, Scopus, ScienceDirect, Wiley Online Library, Google Scholar, and ACS Publications for studies published up to December 2025. Search terms combined “polycystic ovary syndrome/PCOS” with seed-related terms (flaxseed, sesame, pumpkin, sunflower, chia, fenugreek, seed cycling) and nutraceuticals (myo‑inositol, D‑chiro‑inositol, omega‑3 fatty acids, curcumin, N‑acetylcysteine, coenzyme Q10, selenium, vitamin D, alpha‑lipoic acid). Among seeds, flaxseed trials and mixed-seed interventions (pumpkin/sunflower/sesame/flaxseed) have the most direct clinical data in PCOS and have been associated with improvements in insulin resistance indices (e.g., homeostatic model assessment of insulin resistance (HOMA-IR)), inflammatory markers, lipid profiles, and selected reproductive parameters (e.g., luteinizing hormone (LH)/follicle-stimulating hormone (FSH) ratio, ovarian morphology). However, heterogeneity in dosage, duration, and comparators limits firm conclusions. Pumpkin, sunflower, and sesame seeds may have potential mechanistic roles that are supported by human studies in related metabolic conditions, but PCOS-specific trials of individual seeds are still limited. Proposed “seed cycling” protocols have received attention, yet most clinical data are derived from mixed-seed supplementation studies or low-level evidence, such as case reports, and should be considered hypothesis-generating. Among nutraceuticals, inositols, omega‑3 fatty acids, curcumin, and N‑acetylcysteine show comparatively stronger clinical signals in PCOS, primarily for insulin sensitivity and selected endocrine or reproductive outcomes, whereas evidence for micronutrients such as selenium is more context-dependent. Dietary seeds and selected nutraceuticals represent promising adjuncts for PCOS management, especially for cardiometabolic risk modification, but the evidence base is limited by heterogeneity in PCOS phenotyping, interventions, and outcomes. Future trials should standardize diagnostic criteria, dosing, and clinically meaningful endpoints and explore which PCOS phenotypes benefit most from specific seed-based and nutraceutical strategies.

## Introduction and background

Polycystic ovary syndrome (PCOS) is among the most frequent endocrine disorders in women of reproductive age and is strongly associated with multiple cardiometabolic disturbances. Globally, the condition affects roughly 4-20% of women, underscoring its significance as a public-health concern [1,2]. PCOS is defined by the triad of hyperandrogenism, anovulation, and polycystic ovarian morphology, but its clinical presentation extends far beyond the ovaries. Many women suffering from PCOS experience irregular menses, infertility, insulin resistance, obesity, dyslipidaemia, and chronic low-grade inflammation, all of which contribute to long-term risks for type 2 diabetes and cardiovascular disease [3,4].

Conventional management involves lifestyle changes with pharmacological therapy such as oral contraceptive pills, insulin-sensitizing agents, and anti-androgen drugs. While these approaches can reduce symptoms, they mostly do not address the underlying endocrine or metabolic disturbances and may be associated with adverse effects, including gastrointestinal discomfort, weight gain, and fatigue [5,6]. Consequently, there is growing interest in safer, sustainable, and nutrition-based strategies that address the pathophysiology of PCOS more holistically [7-9].

Among emerging approaches, dietary seeds have attracted attention for their dense concentration of bioactive compounds capable of influencing hormonal and metabolic pathways. Flax and sesame seeds provide lignans and phytoestrogens that bind to estrogen receptors and promote the conversion of stronger estrogens (estradiol) into weaker forms (estrone), thereby re-establishing a balanced estrogen-progesterone ratio [4,9,10]. Flaxseed lignans also enhance sex-hormone-binding globulin (SHBG) activity, helping to moderate circulating androgens, while sesame lignans contribute to luteal-phase hormonal balance. Pumpkin seeds, abundant in zinc, support the synthesis of follicle-stimulating and luteinizing hormones, facilitating ovulation; sunflower seeds, rich in vitamin E and selenium, aid progesterone production and protect ovarian tissues from oxidative injury [4,11]. Overall, these seeds appear to modulate key mechanisms related to PCOS, such as ovarian steroidogenesis, gonadotropin signaling, insulin sensitivity, and oxidative stress [4,9,10].

Beyond seeds, a broader range of nutraceutical compounds has been explored for their potential to improve metabolic and reproductive dysfunction in PCOS. Among the most widely studied are the inositol isomers myo-inositol (MI) and D-chiro-inositol (DCI), which enhance insulin sensitivity and help re-establish ovulatory cycles [12,13]. Omega-3 fatty acids improve lipid metabolism and reduce inflammatory activity [14,15], whereas vitamin D and other micronutrients regulate reproductive hormones and glucose homeostasis [16]. Increasing evidence also points to the benefits of curcumin, a polyphenolic compound from turmeric, which down-regulates inflammatory mediators and oxidative stress, thereby improving insulin action [17].

Likewise, resveratrol, N-acetylcysteine (NAC), and coenzyme Q10 have demonstrated antioxidant and anti-inflammatory properties that may improve androgen balance and ovarian performance [18,19].

Although individual studies suggest promising outcomes, existing evidence remains fragmented. Variability in study design, dosage, treatment duration, and clinical endpoints limits clear conclusions regarding the comparative effectiveness of these interventions. A systematic integration of current findings is therefore needed to clarify the mechanistic pathways, therapeutic efficacy, and practical application of seeds and nutraceuticals in PCOS management. Accordingly, this narrative review aims to critically assess and synthesize the available literature on the role of seeds and nutraceutical compounds in the management of PCOS, emphasizing their biochemical mechanisms, clinical outcomes, and potential incorporation into comprehensive dietary and therapeutic frameworks for women with the syndrome.

## Review

Methodology

**Figure 1:**
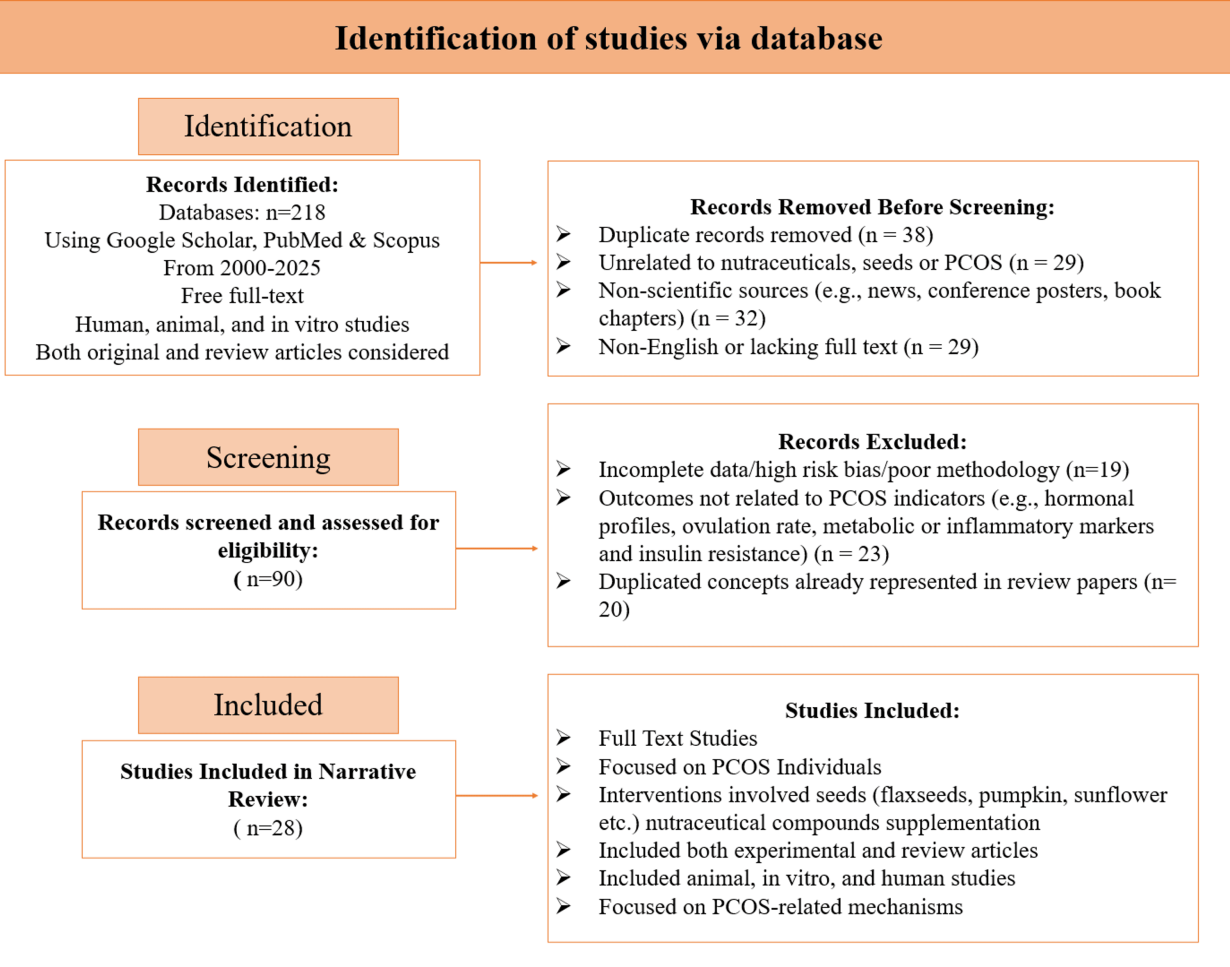
PRISMA flow diagram for literature selection PRISMA: Preferred Reporting Items for Systematic Reviews and Meta-Analyses [20]. PCOS: polycystic ovary syndrome

A comprehensive electronic search was carried out across PubMed, Scopus, Science Direct, the Willey online library, Google Scholar and ACS publications were explored extensively using keywords including Polycystic Ovary Syndrome”, “PCOS”, “seeds”, “flaxseed”, “fenugreek”, “chia seed”, “sesame seed”, “pumpkin seeds”, “bioactive compounds”, “nutraceutical”, “functional foods”, ”, “antioxidants”, “essential fatty acids”, “fibre”, “omega-3 fatty acids”, “vitamins”, and “minerals”. Studies were included if they met the following criteria: (a) women diagnosed with PCOS; (b) assessed the effects of seeds or nutraceutical compounds on metabolic, hormonal, or reproductive outcomes; (c) were randomized controlled trials (RCTs), clinical trials, or observational studies; and (d) were peer-reviewed full-text articles. Exclusion criteria included studies on pharmacological treatments only or lacking PCOS-specific outcomes. Although this is a narrative review, we used a systematic search strategy (Figure 1) adopted from Preferred Reporting Items for Systematic Reviews and Meta-Analyses (PRISMA) guidelines [20].

Figure 1 outlines the selection process for studies retrieved from PubMed, Scopus, and Google Scholar (2000-2025). A total of 218 records were identified, and 28 studies were included in the final narrative synthesis after removing duplicates and irrelevant articles. Both experimental (human, animal, and in vitro) and review studies were considered, focusing on seed-derived and nutraceutical interventions with relevance to PCOS outcomes. Table 1 gives a summary of these studies.

**Table 1:** Summary of all studies FSH: follicle-stimulating hormone; LH: luteinizing hormone; HOMA-IR: homeostatic model assessment of insulin resistance; HDL: high-density lipoprotein; PCOS: polycystic ovary syndrome; DHEA: dehydroepiandrosterone; hsCRP: high-sensitivity C-reactive protein; MI: myo-inositol; DCI: D-chiro-inositol; BMI: body mass index; RCT: randomized controlled trial; RCTs: randomized controlled trials; RI: reflection index; LDL-C: low-density lipoprotein cholesterol; HDL-C: high-density lipoprotein cholesterol; NAC: N-acetylcysteine; ALA: alpha-lipoic acid; IVF: in vitro fertilization; PPAR-γ: peroxisome proliferator-activated receptor gamma; GLUT-1: glucose transporter type 1; LDLR: low-density lipoprotein receptor; MDA: malondialdehyde; ER stress: endoplasmic reticulum stress

Food/ Nutraceutical	Study Design	Intervention	Findings	Author
Flaxseed	RCT Women with PCOS = 70	30 g of milled flaxseed for 12 weeks	Increased levels of FSH hormone and decreased LH/FSH ratio	Najdgholami et al., 2025 [21]
Flaxseed	Women with PCOS = 30	15 g of flaxseed powder with milk for 3 months	Improved menstrual cycle and pregnancy, significant reduction in ovarian volume and number of follicles	Farzana et al., 2015 [22]
Flaxseed	Women with PCOS = 41	30 g per day for 12 weeks	Reduction in body weight, insulin concentration, Homeostatic Model Assessment of Insulin Resistance (HOMA-IR), leptin, and an increase in high-density lipoprotein (HDL)	Haidari et al., 2020 [5]
Flaxseed	Randomized open-label controlled clinical trial, Women with PCOS = 50	Lifestyle modification and 30 g flaxseed powder for 12 weeks	Significant decrease in body weight, liver enzymes, insulin resistance, hepatic fibrosis, and steatosis	Yari et al., 2016 [23]
Pumpkin, sunflower, sesame, and flaxseed	RCT Women with PCOS: 60	Pumpkin, sunflower, sesame, and flaxseed (15 g each powder) with portion-controlled diets for 90 days	Significant decrease in FSH and LH hormones	Rasheed et al., 2023 [9]
Flaxseed, sunflower, pumpkin, and sesame seed	Case Study: 29-year-old female with PCOS	Flaxseed & pumpkin seed during the follicular phase and sunflower & sesame during the luteal phase for 6 months	Regular menstrual cycle, reduction in body mass index, and normalised hormonal levels	Dhamija et al., 2025 [24]
Flaxseed, pumpkin, sunflower, sesame	Women with PCOS: 30	10 g of seeds four times per day for 3 months	Significant reduction in BMI and improve menstrual cycle	Ajith & Meera, 2024 [10]
Selenium	RCT Women with PCOS (18 –40-year-old) = 64	200 μg selenium daily (n=32) and placebo (n=32) for 8 weeks.	Pregnancy rate in the selenium group was higher than in the placebo group. Significantly decreased serum dehydroepiandrosterone (DHEA) levels, hirsutism, serum high-sensitivity C-reactive protein (hsCRP), and increased pregnancy rate	Razavi et al., 2016 [25]
Myo‐inositol (MI) and D‐chiro‐inositol (DCI)	RCT Women with PCOS = 46	MI plus DCI in a 40:1 ratio for 6 months	Significant reduction of LH, free testosterone, fasting insulin, and HOMA index, significant increase of 17‐beta‐Estradiol levels	Benelli et al., 2016 [12]
Vitamin E	Retrospective cohort clinical trial Women with PCOS: 321	Oral Vitamin E 100 g/ day during the follicular and luteal phases	No significant differences in ovulation rate, clinical pregnancy rate, and ongoing pregnancy rate	Chen et al., 2020 [26]
Vitamin E	RCT Girls = 278	200 units of vitamin E twice a day for 4 months	Vitamin E helps relieve the pain of dysmenorrhea and reduces menstrual blood loss	Ziaei et al., 2005 [27]
Amla	RCT Normal and Diabetic Participants	4 g Amla powder per day for 21 days	Significant reductions were observed in fasting and postprandial glucose, total cholesterol, and triglyceride levels	Akhtar et al., 2011 [28]
Amla	Randomised, double-blind, placebo-controlled. Participants = 50	Amla aqueous extract in capsule form, that is 250 mg and 500 mg twice daily for 12 weeks	Significant reduction in the reflection index (RI), total cholesterol, and triglycerides, and a significant increase in HDL. Improved Oxidative stress markers while hsCRP levels decreased significantly	Usharani et al., 2019 [29]
Date	RCT of 100 Type 2 Diabetes participants	Three dates daily for 16 weeks	Improved lipid profile with decreased total cholesterol and increased HDL	Alalwan et al., 2020 [30]
Curcumin	Randomised, double-blind, placebo-controlled clinical trial: Women with PCOS = 60	Curcumin – 500 mg/day for 12 weeks	Significant reduction in BMI, total cholesterol, triglycerides, insulin resistance, and increased insulin sensitivity and HDL levels	Jamilian et al., 2020 [31]
Curcumin (Review)	RCTs Women with PCOS=296	80 mg to 500 mg of curcumin for 6-12 weeks	Significant improvements in BMI, fasting insulin, HOMA-IR, total cholesterol, triglycerides, LDL-C, and an increase in HDL-C. Improved insulin sensitivity and lipid profile	Abdelazeem et al., 2022 [32]
Curcumin (Review)	RCTs Women with PCOS = 79	500 mg of curcumin for 6-12 weeks	Significant reduction in fasting blood glucose, fasting insulin, HOMA-IR, total cholesterol, triglycerides, and LDL-C; significant increase in HDL-C and insulin sensitivity	Chien et al., 2021 [33]
Myo-inositol (Review)	RCT Women with PCOS	2-4 g of myo-inositol for 3 to 6 months	Significant improvement in ovulatory function, menstrual regularity, insulin sensitivity, and reduction in fasting insulin, BMI, and androgen levels	Fitz et al., 2024 [34]
Omega-3 fatty acids	Women with PCOS = 185	Dietary intake and serum levels of omega-3 fatty acids were measured	Higher omega-3 fatty acid levels were significantly associated with lower insulin resistance (HOMA-IR), reduced BMI, waist circumference, and improved body composition	Lu et al., 2022 [35]
N-acetylcysteine (Review)	RCT Women with PCOS = 2515	N-acetylcysteine 1200-1800 mg/day for 8-24 weeks	Significant improvements in fasting insulin, HOMA-IR, total testosterone, LH/FSH ratio, ovulation rate, and menstrual regularity	Viña et al., 2025 [36]
N-acetylcysteine (NAC) (Review)	RCT Women with PCOS = 869	1200-1800 mg/day for 6–24 weeks	Significantly reduced fasting glucose, fasting insulin, total cholesterol, and triglycerides	Liu et al., 2023 [37]
Alpha-lipoic acid (ALA) (Review)	RCTs Women with PCOS	600-800 mg/day for 6-24 weeks	Significantly reduced fasting blood sugar and HOMA-IR, indicating improved insulin sensitivity and glycemic control in women with PCOS	Abu-Zaid et al., 2024 [38]
Coenzyme Q10 and Vitamin E	RCT Women with PCOS =86	Coenzyme Q10 (200 mg/day) and Vitamin E (400 IU/day) for 8 weeks	Significantly reduced fasting insulin, HOMA-IR, total testosterone, LH, and LH/FSH ratio; improved insulin sensitivity and lipid profile	Izadi et al., 2019 [39]
Selenium	RCT 40 infertile females with PCOS aged between 18 and 40 years	200 μg/day selenium for 8 weeks	Selenium significantly decreased fasting glucose, fasting insulin, and HOMA-IR and significantly increased the quantitative insulin sensitivity check index. It also reduced malondialdehyde (MDA) levels, indicating improved oxidative stress status	Modarres et al., 2022 [40]
Selenium	Randomized double-blind, placebo-controlled trial of PCOS women for IVF =40	200 μg selenium for 8 weeks	Significant reductions in fasting glucose, insulin, and HOMA-IR; increased insulin sensitivity. Selenium upregulated PPAR-γ and GLUT-1 and downregulated LDLR gene expression. Reduced malondialdehyde (MDA) levels	Modarres et al., 2018 [41]
Gelatinized Brown Rice Extract	In vitro experimental study Participants: Type 2 diabetes patients	Treatment of human liver cells (HepG2) with gelatinized brown rice extract	Its strong antioxidant activity, inhibition of α-glucosidase, and upregulation of insulin-signaling and glucose-transport genes may help reduce insulin resistance and improve glucose metabolism—key underlying mechanisms in PCOS pathophysiology	Kang et al., 2024 [42]
N-acetylcysteine (Animal study)	Randomized controlled clinical trial of female mice with PCOS = 86	N-acetylcysteine (NAC) – 600 mg three times daily for 12 weeks	Significant improvement in fasting insulin, HOMA-IR, total testosterone, LH/FSH ratio, and lipid profile; enhanced ovulation induction rate and menstrual regularity	Fang et al., 2024 [43]
Sesame Oil (Animal Study)	RCT Female rats with PCOS = 28	2 ml/kg body weight/day for 21 days	Improved hormonal, metabolic, inflammatory, and endoplasmic reticulum stress (ER stress) levels	Elshamy et al., 2023 [44]

Flaxseeds

Flaxseed (*Linum usitatissimum*) has emerged as a promising functional food for managing PCOS, owing to its rich content of lignans (phytoestrogens), α-linolenic acid (omega-3 fatty acid), fiber, and phytosterols such as β-sitosterol and stigmasterol [23]. Human clinical trials demonstrate that flaxseed supplementation significantly improves reproductive endocrine function in women with PCOS. In a randomized controlled trial, Najdgholami et al. [21] reported that daily flaxseed intake for 12 weeks led to a significant increase in follicle-stimulating hormone (FSH) and a reduction in the luteinizing hormone (LH)/FSH ratio, suggesting restoration of ovulatory function and hormonal balance. Similarly, Farzana et al. [22] observed that flaxseed supplementation decreased ovarian volume and follicle number while improving menstrual cycle regularity. Metabolic outcomes have also been favourably influenced by flaxseed intake. In a clinical trial, Haidari et al. [5] found that 30 g/day of ground flaxseed combined with lifestyle modification resulted in a reduction in insulin resistance (homeostatic model assessment of insulin resistance (HOMA-IR)), improvement in lipid profile, and lowered inflammatory markers (high-sensitivity C-reactive protein (hsCRP)) compared to controls. These results are supported by Rasheed et al. [9], who demonstrated that a mixed-seed regimen including flaxseed, sunflower seeds, pumpkin seeds, and sesame seeds improved insulin sensitivity and reduced hormonal disturbances in women with PCOS. A recent in silico and in vitro study suggested that flaxseed bioactives can influence insulin receptor signalling and inflammatory cascades relevant to PCOS pathophysiology. Furthermore, Zafar et al. [11] highlighted flaxseed's role within seed cycling protocols, particularly during the follicular phase, where its phytoestrogenic lignans support estrogen modulation and cycle regularity.

Pumpkin seeds

Pumpkin seeds (*Cucurbita pepo*) are nutritionally dense seeds rich in zinc, magnesium, unsaturated fatty acids (omega-3 and omega-6), phytosterols (β-sitosterol, stigmasterol), antioxidants (tocopherols, carotenoids, polyphenols), and amino acids such as arginine and tryptophan, all of which exert diverse metabolic and endocrine effects relevant to the management of PCOS [45,46]. Zinc acts as a crucial cofactor for numerous enzymes involved in insulin synthesis, glucose metabolism, and steroid hormone regulation, while magnesium contributes to insulin receptor signaling and oxidative stress reduction, both critical for alleviating insulin resistance and hyperandrogenism commonly observed in PCOS [46,47]. The unsaturated fatty acids (ω-3 and ω-6) and phytosterols in pumpkin seeds enhance membrane fluidity, modulate inflammatory mediator synthesis, and influence adipokine and androgen receptor signaling, thereby supporting lipid regulation, estrogen metabolism, and ovarian function [45,47]. Antioxidant compounds such as tocopherols, carotenoids, and polyphenols scavenge reactive oxygen species and limit lipid peroxidation, which can otherwise impair follicular maturation and insulin sensitivity [45].

Additionally, amino acids like arginine promote nitric oxide synthesis, improving vascular perfusion and metabolic homeostasis, while tryptophan contributes to neuroendocrine regulation and stress response, both relevant to PCOS symptom control [46]. In integrative seed-cycling protocols, Zafar et al. found that pumpkin seeds are typically consumed during the follicular phase, paired with flaxseed, to promote estrogen metabolism, ovulatory preparation, and cycle regularity [11]. Clinical evidence from a randomized controlled trial by Rasheed et al. found that combined seeds (pumpkin, flax, sesame, sunflower) have shown a significant improvement in insulin sensitivity, lipid profile, and hormonal balance, including reductions in LH/FSH ratio and androgen levels [9]. Emerging reports and case studies also suggest that regular pumpkin seed intake contributes to lower testosterone, improved glucose-insulin ratio, and better BMI control in women with PCOS, likely through the synergistic actions of its micronutrients and phytochemicals [24,47].

Sunflower seeds

Sunflower seeds (*Helianthus annuus*) are a nutrient-dense source of vitamin E, selenium, healthy unsaturated fatty acids, and trace zinc, making them relevant to nutritional strategies for PCOS due to their potential to influence oxidative stress, inflammation, and reproductive hormone balance. Oxidative stress is a recognized contributor to the pathophysiology of PCOS, promoting insulin resistance, chronic inflammation, and ovarian dysfunction [26,48]. Vitamin E, the predominant antioxidant in sunflower seeds, has been evaluated in randomized controlled clinical trials primarily in the context of menstrual health: supplementation studies demonstrate that vitamin E significantly reduces pain severity and duration in primary dysmenorrhea, an outcome linked to its inhibition of prostaglandin synthesis and attenuation of lipid peroxidation [27,49]. Furthermore, systematic reviews suggest that vitamin E intake, whether as a supplement or from food sources, can improve lipid profiles, insulin resistance, reproductive hormone ratios, and oxidative stress markers in women with PCOS, reinforcing the mechanistic relevance of sunflower seed antioxidants [50].

Selenium, another micronutrient abundant in sunflower seeds, acts as a cofactor for glutathione peroxidase and other redox-regulating enzymes; clinical evidence indicates that selenium supplementation may modulate inflammatory and oxidative biomarkers in women with PCOS, although results are variable and context-dependent [25]. Healthy unsaturated fats in sunflower seeds also align with evidence showing that dietary vitamin E and omega-3 fatty acids improve insulin sensitivity, lipid metabolism, and inflammatory gene expression (e.g., peroxisome proliferator-activated receptor gamma (PPAR-γ), interleukin 8 (IL-8), tumor necrosis factor alpha (TNF-α)) in PCOS cohorts [51]. In integrative approaches such as seed-cycling protocols, sunflower seeds are recommended during the luteal phase to support progesterone production, antioxidant defense, and menstrual comfort via their vitamin E and selenium content [11,52]. While direct randomized clinical trials investigating sole sunflower seed intake in PCOS are not yet published, interventional seed-cycling studies that include sunflower seeds alongside flaxseed, pumpkin seed and sesame seed report improved hormonal profiles, insulin sensitivity and ovarian morphology in women with PCOS, implying potential benefits from the bioactive constituents of sunflower seeds when consumed as part of a structured dietary intervention [9].

Sesame seeds

Sesame seeds (*Sesamum indicum*) are nutrient-dense seeds providing lignans (sesamin, sesamolin), vitamin E, calcium, zinc, and unsaturated fatty acids, all of which contribute to antioxidant, anti-inflammatory, and potential hormone-modulating effects relevant to polycystic ovaries [53]. The major sesame lignans sesamin and sesamolin exhibit phytoestrogen-like activity, influencing estrogen and progesterone metabolism through modulation of steroidogenic enzymes and hepatic estrogen clearance [54,55]. Mechanistically, these lignans activate PPAR-α and AMP-activated protein kinase (AMPK), enhance fatty acid oxidation, suppress nuclear factor kappa-light-chain-enhancer of activated B cells (NF-κB)-mediated inflammation, and increase antioxidant enzyme activity, thereby improving lipid and glucose metabolism [55,56]. Clinical studies and systematic reviews in metabolic and cardiovascular contexts have demonstrated that sesame consumption or sesamin supplementation can significantly reduce total cholesterol, low-density lipoprotein cholesterol (LDL-C), and systolic blood pressure and improve oxidative stress and inflammatory markers. It has also been found that sesame intake led to reductions in fasting glucose, glycated hemoglobin (HbA1c), and C-reactive protein (CRP), supporting its value in metabolic-syndrome-type states that overlap with PCOS [56-58].

Although direct randomized controlled trials on isolated sesame seed intake in women with PCOS are limited, emerging interventional evidence from combined-seed protocols incorporating sesame alongside flaxseed, sunflower, and pumpkin seeds shows promising effects. In a 12-week clinical trial, women with PCOS receiving a daily mixed-seed supplement (15 g of each seed powder/day) experienced improvements in FSH, LH, and progesterone levels, reduced ovarian volume, follicle number, better BMI, and insulin sensitivity compared with controls [9]. These findings align with the seed-cycling framework in which sesame seeds are consumed during the luteal phase together with sunflower seeds to support progesterone production and menstrual comfort [11]. Beyond reproductive endpoints, both animal and human studies indicate that sesame seed and lignan consumption enhances antioxidant capacity, improves hepatic lipid metabolism, and lowers oxidative stress biomarkers, all of which are beneficial in addressing the metabolic inflammation and insulin resistance characteristic of PCOS [44,58].

Finger millet (ragi)

Finger millet (*Eleusine coracana*), commonly known as ragi, is a nutrient-dense cereal and a recognized nutraceutical compound rich in dietary fiber (~15-20%), polyphenols, flavonoids, and minerals such as calcium and iron, which contribute to its potential metabolic benefits, including modulation of glucose metabolism and weight management [59]. The dietary fiber and phenolic compounds in ragi slow carbohydrate digestion and glucose absorption, leading to lower postprandial glycemic responses and attenuated insulin spike effects highly relevant to improving insulin sensitivity in women with PCOS, where insulin resistance is a central pathological feature [60]. It was also observed that regular finger millet consumption significantly lowers fasting and postprandial blood glucose and reduces HbA1c compared with equivalent refined grain diets, indicating that millet-based diets may favorably influence glucose homeostasis in metabolic disorders relevant to PCOS [60,61].

Beyond glycaemic control, the prebiotic fiber and polyphenols in ragi are metabolized by gut microbiota into short-chain fatty acids (SCFAs) such as butyrate and propionate, which improve insulin sensitivity, reduce systemic inflammation, and modulate lipid metabolism pathways commonly dysregulated in PCOS [62]. The high fiber content of finger millet also enhances satiety and reduces caloric intake, supporting weight management, a key lifestyle strategy for improving insulin resistance and reproductive outcomes in PCOS [63,64].

Indian gooseberry (amla)

Amla (*Emblica officinalis*), a recognized nutraceutical compound, is rich in vitamin C, polyphenols, and flavonoids that confer antioxidant and anti-inflammatory effects relevant to metabolic disorders such as PCOS [65]. Clinical evidence from a systematic review and meta-analysis demonstrates that amla supplementation leads to significant reductions in fasting blood glucose, LDL-C, total cholesterol, and triglycerides and increases in high-density lipoprotein cholesterol (HDL-C) in adults with cardiometabolic risk factors, supporting its role in improving insulin sensitivity and lipid metabolism [66]. In a randomized controlled trial, Akhtar et al. (2011) reported that in normal and type 2 diabetic volunteers, the daily intake of amla fruit powder for 21 days significantly lowered fasting and postprandial blood glucose and improved total cholesterol and triglyceride levels compared with baseline [28]. Another clinical study by Usharani et al (2019) showed that a standardized aqueous extract of amla significantly improved lipid profile, endothelial function, markers of oxidative stress, and systemic inflammation in subjects with metabolic syndrome compared with placebo [29].

Although direct randomized controlled trials specifically in women with PCOS are currently limited, the existing human clinical evidence supports that amla positively influences glycemic control, lipid profiles, and inflammation, key aspects of PCOS pathophysiology [66]. In addition, mechanistic and preclinical research further indicates that amla’s bioactive phytochemicals enhance antioxidant defenses and may modulate pathways involved in insulin resistance and dyslipidemia, although these mechanisms require further confirmation in PCOS populations [67]. Taken together, clinical and mechanistic evidence support the potential of amla as a nutraceutical agent for improving metabolic parameters commonly impaired in PCOS.

Dates

Dates (*Phoenix dactylifera*) are nutrient-rich fruits high in natural sugars combined with dietary fiber, potassium, polyphenols, and flavonoid antioxidants, which help slow carbohydrate absorption and modulate oxidative stress, making them a functional food with potential metabolic benefits [68]. Systematic reviews and clinical trials indicate that moderate date consumption does not worsen glycemic control and may improve lipid metabolism, including reductions in total cholesterol and LDL cholesterol and increases in HDL cholesterol in adults with metabolic risk factors [69]. In type 2 diabetes patients, daily modest date intake (three dates per day) did not adversely affect fasting glucose or HbA1c compared with other snacks, confirming glycemic safety over 16-week intervention periods [30]. Similarly, Ajwa date pit powder supplementation in adults with hyperlipidemia showed reductions in body weight, BMI, fat mass, total cholesterol, and LDL, suggesting beneficial effects on obesity and lipid parameters, which are commonly associated with PCOS [70]. These human studies support that dates are metabolically safe and may contribute to improved glycemic and lipid regulation in conditions related to insulin resistance.

Further studies support the metabolic benefits of date bioactives. Dates and their seeds are rich in polyphenols and flavonoids, particularly phenolic acids, quercetin, and luteolin, that exhibit strong antioxidant and anti-inflammatory activity, enhance endogenous antioxidant enzymes such as superoxide dismutase and catalase, and reduce oxidative stress markers involved in metabolic dysregulation seen in PCOS [71]. In hypercholesterolemic rat models, Ajwa date extracts significantly lowered total cholesterol, LDL, very-low-density lipoprotein (VLDL), and triglycerides while increasing HDL, demonstrating the lipid-modulating potential of date-derived fiber and bioactive compounds [72]. Moreover, in vitro studies with polyphenol-rich date seed extracts show increased glucose uptake, upregulation of glucose transporter type 4 (GLUT-4) and AMPK signaling, and inhibition of adipocyte differentiation mechanisms that support improved insulin sensitivity and weight regulation relevant to PCOS management [73].

Jaggery

Jaggery is an unrefined sweetener from sugarcane or palm sap, retaining trace minerals such as iron, calcium, magnesium, potassium, and antioxidant polyphenols, which give it higher nutritive and functional value than refined sugar [74]. Analytical studies show that jaggery has higher phenolic content and antioxidant activity, suggesting potential free radical scavenging properties that may influence oxidative stress, a factor implicated in insulin resistance and metabolic disturbances in PCOS [75].

While jaggery contains antioxidants, minerals, and other micronutrients that contribute to its nutraceutical profile, these compounds exist alongside a high concentration of rapidly digestible sugars, meaning that the nutrient benefits are unlikely to counteract its impact on postprandial glycemia unless intake is limited [76]. Dietary fiber has been shown to slow glucose absorption and reduce rapid postprandial glucose rises when carbohydrates are ingested, likely due to increased intestinal viscosity and delayed gastric emptying [77]. Similarly, higher protein intake can alter postprandial glucose responses and support more stable glucose levels when combined with carbohydrate intake [78,79]. Thus, in PCOS diets, jaggery should be consumed in small amounts and ideally paired with fiber-rich or protein-rich foods that slow carbohydrate digestion and absorption.

Inositol (myo‑inositol and D‑chiro‑inositol)

Inositols, particularly myo‑inositol (MI) and D‑chiro‑inositol (DCI), are sugar‑like compounds considered nutraceuticals that support insulin signalling and ovarian function in women with PCOS. Meta‑analyses of randomized controlled trials show MI reduces fasting insulin, while MI + DCI in a physiological 40:1 ratio improves menstrual regulation, ovulation rates, and endocrine markers in PCOS patients, with effects similar to or complementary to metformin for metabolic and reproductive parameters. Trials also demonstrate significant improvements in ovarian volume and quality of life measures with MI + DCI supplementation compared with standard therapy alone, supporting its role in enhancing insulin sensitivity and reproductive outcomes [34].

Curcumin

Curcumin, the main polyphenolic compound in turmeric, has demonstrated antioxidant and anti‑inflammatory effects that translate into improved metabolic parameters in women with polycystic ovary syndrome [80]. Randomized clinical trials show that curcumin supplementation for 12 weeks significantly reduces fasting glucose, fasting insulin, and HOMA‑IR, and improves insulin sensitivity compared with placebo, indicating enhanced glycemic control and insulin resistance [32,33]. Additionally, in a randomized controlled trial (RCT) of women with PCOS receiving metformin, adding curcumin was associated with greater reductions in fasting insulin and HOMA‑IR and improvements in HDL cholesterol and total cholesterol than metformin alone [81]. Other randomised control trial reports that curcumin alone leads to better serum insulin levels and insulin sensitivity indices versus placebo, although effects on some lipid markers and hyperandrogenism vary [31]. Systematic reviews and meta‑analyses of these RCTs confirm curcumin’s benefits on glycemic control (fasting glucose, insulin, HOMA‑IR) and select cardiometabolic risk factors in PCOS, supporting its use as a nutraceutical adjunct.

Omega‑3 fatty acids

Omega‑3 polyunsaturated fatty acids (PUFAs), particularly eicosapentaenoic acid (EPA) and docosahexaenoic acid (DHA) from fish oil or plant sources, are supported by systematic reviews and meta-analyses of randomized controlled trials as effective nutraceuticals for improving metabolic status with significant reductions in insulin resistance and total cholesterol in women with polycystic ovary syndrome. Supplementation with omega‑3 PUFAs has been shown to significantly reduce insulin resistance, as measured by HOMA‑IR, fasting insulin, triglycerides, total cholesterol, and LDL cholesterol, while increasing adiponectin, highlighting improvements in insulin sensitivity and lipid metabolism compared with controls [82,83]. Some studies also demonstrate that long‑chain omega‑3 PUFAs are negatively correlated with HOMA‑IR and body fat measures in PCOS patients, emphasizing their beneficial effects on body composition and overall metabolic function [35]. With consistent improvements in insulin and lipid indices, support omega‑3 as a beneficial adjunct to lifestyle and pharmacologic interventions in PCOS. Observational analyses in PCOS also report associations between omega‑3 measures and insulin resistance/body composition, though these are not substitutes for randomized evidence [36].

Alpha‑lipoic acid (ALA)

Alpha lipoic acid (ALA) is a sulfur-containing mitochondrial antioxidant and enzyme cofactor. It is a potential antioxidant and has been evaluated in women with PCOS. Systematic reviews and meta‑analyses of randomized clinical trials indicate that ALA supplementation significantly improves glycemic control by reducing fasting blood glucose and HOMA‑IR compared with controls, supporting its role in enhancing insulin sensitivity [38]. A review study suggests that ALA in combination with inositol and other compounds may further optimize hormonal balance and metabolic outcomes in PCOS, though dedicated large randomized controlled trials of ALA alone remain limited [84].

N‑acetyl cysteine

N‑acetylcysteine (NAC), a well-known antioxidant and precursor of glutathione, has been investigated in several clinical trials and systematic reviews among women with PCOS. NAC supplementation significantly enhances ovulation induction efficacy and increases pregnancy rates in women undergoing ovulation induction protocols, while also improving endocrine and metabolic parameters, including reductions in LH/FSH ratios, improvements in insulin sensitivity, and enhanced antioxidant enzyme activity compared with control groups [36,43]. Meta-analyses show that NAC significantly increases progesterone levels and endometrial thickness - parameters linked to improved reproductive outcomes - and may also reduce oxidative stress markers, providing mechanistic support for its insulin-sensitizing and antioxidant effects [36,37].

Coenzyme Q10 (CoQ10)

Coenzyme Q10 (CoQ10), a lipid‑soluble antioxidant involved in mitochondrial energy metabolism and free radical scavenging, has emerging clinical evidence showing that supplementation can improve metabolic and endocrine indices, including reductions in fasting plasma glucose, insulin, and HOMA‑IR, and may enhance ovarian response and pregnancy outcomes when used as an adjuvant in ovulation induction protocols [39,85].

Selenium (Se)

Selenium, an essential trace mineral and cofactor for antioxidant selenoproteins (e.g., glutathione peroxidases, thioredoxin reductases), has been studied in women with polycystic ovary syndrome (PCOS) for its effects on oxidative stress and metabolic parameters. Randomized trials show that selenium supplementation (200 μg/day) reduces fasting glucose, insulin, HOMA‑IR, and oxidative stress markers (malondialdehyde (MDA)) and increases insulin sensitivity indices compared to placebo [40,41]. Selenium may also modulate gene expression related to insulin and lipid metabolism in PCOS patients, suggesting potential cellular metabolic benefits [41]. However, systematic reviews indicate that while selenium consistently improves total antioxidant capacity, its effects on BMI, lipids, and hormonal parameters are variable, highlighting that evidence for broader metabolic benefits remains mixed [86].

**Figure 2:**
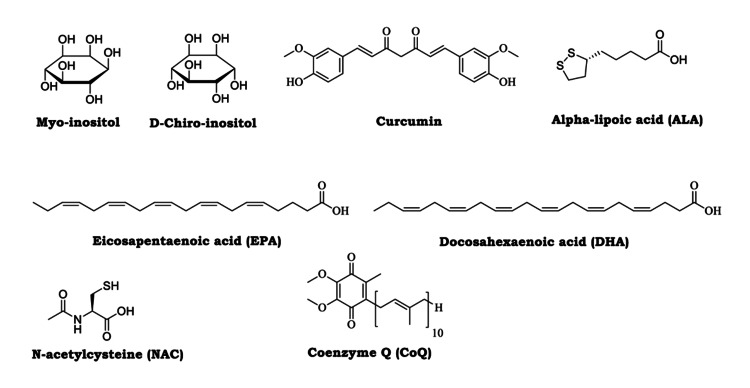
Chemical structures of selected nutraceutical and antioxidant compounds commonly investigated in the management of PCOS. Original figure created with ChemDraw software (Revvity Signals, Waltham, USA). PCOS: polycystic ovary syndrome

**Figure 3:**
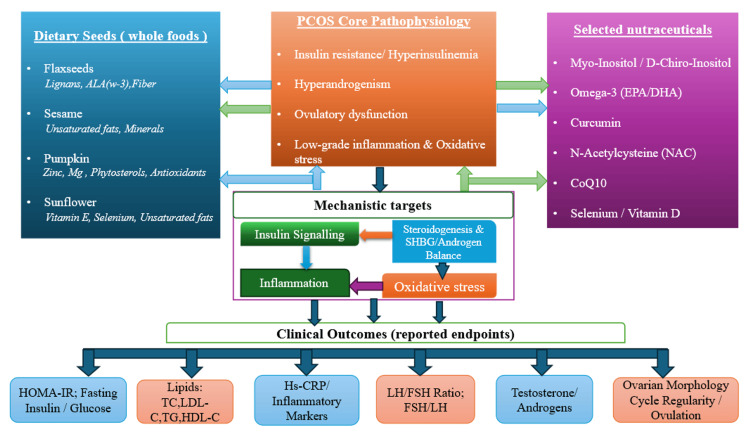
Conceptual framework of dietary seeds and selected nutraceuticals in PCOS Original figure created with PowerPoint software (Microsoft Corporation, Redmond, USA). PCOS: polycystic ovary syndrome; ALA: alpha-linolenic acid; w-3: omega-3; Mg: magnesium; EPA: eicosapentaenoic acid; DHA: docosahexaenoic acid; NAC: N-acetylcysteine; CoQ10: coenzyme Q10; SHBG: sex hormone-binding globulin; HOMA-IR: homeostatic model assessment of insulin resistance; TC: total cholesterol; LDL-C: low-density lipoprotein cholesterol; TG: triglycerides; HDL-C: high-density lipoprotein cholesterol; Hs-CRP: high-sensitivity C-reactive protein; LH: luteinizing hormone; FSH: follicle-stimulating hormone

Figure 2 shows the chemical structures of selected nutraceutical and antioxidant compounds commonly investigated in the management of PCOS. Figure 3 presents the conceptual framework of this review.

Definitions

Functional foods are novel foods that have been formulated so that they contain substances or live microorganisms that have a possible health-enhancing or disease-preventing value, and at a concentration that is both safe and sufficiently high to achieve the intended benefit [87]. Nutraceuticals are food-derived products or food components, including dietary supplements and bioactive constituents, that confer health benefits beyond basic nutrition and may help prevent or manage disease [88].

## Conclusions

Emerging evidence suggests that therapeutic dietary modifications combined with lifestyle modifications can significantly improve metabolic and reproductive outcomes in women with polycystic ovary syndrome (PCOS). Regular consumption of seeds, nuts, and nutraceutical compounds provides fiber, antioxidants, polyunsaturated fatty acids, and bioactive molecules that enhance insulin sensitivity, lipid metabolism, and reduce oxidative stress. Clinical studies indicate that daily intake of 25-30 g of seeds can achieve measurable metabolic benefits. However, consuming this amount through whole foods alone is often impractical due to taste, convenience, and dietary habits. A potential future direction can be innovations such as multi-seed and nutraceutical-enriched nutri-bars, powders, fortified snacks, and *namkeen*s, which offer convenient, standardized, and bioavailable delivery formats. Coupling these dietary strategies with regular exercise, yoga, or structured workouts further improves insulin sensitivity, hormonal balance, and cardiovascular health, supporting holistic PCOS management. Such integrated approaches facilitate adherence, enable precision nutrition, and target both metabolic and reproductive dysfunction effectively. Future research should focus on optimizing functional food formulations, dose, and long-term efficacy, along with lifestyle modifications to maximize benefits for women with PCOS.
